# Military Medicine at Waterloo

**Published:** 1917-03-03

**Authors:** 


### MILITARY MEDICINE AT WATERLOO.

The later Napoleonic wars, being the campaigns most like the present European strife, have a new interest these last two years. To refresh knowledge of them many great writers may be resorted to, for naturally such a subject few have missed. Of course, the French outnumber the others. Byron, Balzac, Victor Hugo, Stendhal, Tolstoi, Thackeray, Hardy—these seven form a constellation of stars of greater and greatest magnitude in whose light Europe a century dead appears again ghost-like, but yet like life. For the present prosaic purpose, however, one is better served by smaller artists. The usual pleasant homeliness of Erckmann-Chatrian, which in "The Conscript" and "Waterloo" becomes a tragic homeliness, gives just the kind of detail one requires for estimating what it meant to peasants to have to fight to the death without the succour of bacteriology, hygiene, and scientific surgery.

What heightens the contrast between then and now in military medicine is that in so many other war matters there is no difference at all. There are the officers who were underlings and the privates who were gentlefolks. There are tribunals and examining doctors over-ruling excuses. There is getting into munition work, and subsequent "combing out" from the same, the sub-malingerers not finding the first experience of active service so bad after all. The scene is often the same—Verdun, Longwy, the Sambre, heavy Flanders rain, street fighting in Belgian villages. There is the *Gazette* with its wrong forecast, and food at balloon prices. There is, finally, the speech of his martial worship the Mayor, who has chosen the task of keeping the home camp-fires burning.

"Come—courage!" he would cry to them; and then he talked of the Greeks and Romans, and how they used to fight for their country.

"I thought as I listened to him—"If you think that so fine, why don't you go and join yourself?"

But from the sanitary point of view the tale seems old-fashioned. Retreating troops, themselves decimated by typhus, bring the disease into Alsace, where, out of every hundred villagers falling ill, a dozen at the most recover. In the hospital near Leipzig the beds almost touch; the great room is filled with groans, oaths, and moanings of hundreds of wounded men; not to catch the hospital fever is being lucky.

Anæsthetics are unknown, of course; and, what is curious, men at the field hospital who stand an amputation without flinching will faint, when back in bed, at catching sight of their own severed limb at the top of the pile. The most terrible impression of all in the Waterloo rout is that of the faint, despairing glances of wounded in ambulance wagons abandoned by the drivers, who had cut the traces and made off. To be sure, we still lose lives from septic wounds, but there are no such horrors now as those which used to await the European private soldier a century ago. They may, in the telling, be slightly exaggerated, for the narrator seems to take a democratic view of national campaigns, but, with every allowance for that, the difference is vast. The main base hospital at Halle, mentioned above, would not be nearly as large as one of the converted lunatic asylums near large towns that now fill up with wounded after every movement on the Western Front. Yet its surgeons "found" seven or eight dead every morning, whereas in the latter a mortality of one a day is rarely exceeded. And, of course, there has been so little epidemic disease that among the Canadian troops, for instance, who, as regards Europe, must be unacclimatised, sickness accounted for only 1 per cent. of the causes of disablement.

One is struck, then, by this contrast: that whereas human nature is unaltered, and weapons not altered out of recognition—explosives have become "higher," warfare has become aerial, the bayonet remains very important—the means of preservation of life, of healing and alleviation, have been improved as by a transformation. Has this fact, one asks, been duly recognised? When the next Lister appears, will it be honouring him adequately to admit him to equality with successful business men and brewers?

